# Human gastric extracellular matrix hydrogels maintain organoid growth and reduce gene expression associated with stemness and inflammation

**DOI:** 10.1088/1748-605X/ae943e

**Published:** 2026-08-17

**Authors:** Michelle D Cherne, Jasper L Gattiker, Katrina N Lyon, Daniella Russell, Thomas A Sebrell, Alexis Burcham-Carter, Ava P Richard, Eli N Mathew, Samuel G Mackintosh, Andrew J Gorzalski, Diane Bimczok

**Affiliations:** 1 Department of Microbiology and Cell Biology, Montana State University, Bozeman, MT, United States of America; 2 Department of Biomedical Engineering and Science, Florida Institute of Technology, Melbourne, FL, United States of America; 3 Burrell College of Health Sciences, Melbourne, FL, United States of America; 4 Department of Biochemistry and Molecular Biology, University of Arkansas for Medical Sciences, Little Rock, AR, United States of America; 5 Nevada Genomics Center, University of Nevada at Reno, Reno, NV, United States of America

**Keywords:** decellularized tissue, human gastric organoid, single cell RNA sequencing, gastric stroma

## Abstract

Human gastric organoids (HGOs) are 3D cultures of primary gastric epithelial cells that serve as in vitro models for gastric epithelial physiology, development, and disease. The extracellular matrix (ECM) material that organoids are maintained in provides crucial signals to the epithelial cells, regulating their function and growth. We here describe the application of human gastric ECM (hgECM) for HGO cultures, to provide a tissue- and species-specific growth matrix. We prepared hgECM from human gastric tissue by decellularization and then compared HGO growth, differentiation, and function in hgECM and in the traditionally used Matrigel. The composition, structure, and rheological properties of the hgECM were determined using tandem mass spectrometry, scanning electron microscopy, and rheometry. Transcriptional profiles of HGOs grown in hgECM and Matrigel were compared using single cell RNA sequencing (scRNAseq). Proteome analysis showed that hgECM was rich in collagens, whereas Matrigel was predominantly composed of glycoproteins such as laminin. The structure of hgECM was fibrous, befitting its collagen composition, while Matrigel was porous. The viscosity and stiffness of hgECM were found to be lower than Matrigel. HgECM supported robust growth of HGOs. ScRNAseq showed that cell type composition of HGOs in each ECM was comparable. However, HGOs in hgECM had reduced expression of genes associated with stem cells, epithelial-mesenchymal transition, stress, and inflammation compared to HGO grown in Matrigel. Conversely, HGO culture in hgECM increased expression of gastric tissue-specific genes and of genes associated with growth and metabolism. HgECM hydrogels were highly compatible with HGO culture, and improved homeostatic functions of HGOs, including growth, differentiation, and reduced inflammation. HgECM may serve as a tissue- and species-specific growth matrix for HGO culture to improve the physiological relevance of organoid models.

**Abbreviations**
HgECM: human gastric extracellular matrix
HGO: human gastric organoid
ECM: extracellular matrix

## 1. Introduction

Organoids are multicellular cultures derived from adult or pluripotent stem cells. Due to this stem cell origin, organoids develop differentiated cell types and maintain organ-specific functions and microenvironment, making them excellent experimental models for wide ranging applications from developmental biology to patient-specific disease modeling [1, 2]. To support their three-dimensional structure, organoids must be embedded in an ECM. Traditionally, Matrigel, a proprietary preparation of laminin-rich ECM derived from the Engelbreth–Holm–Swarm mouse tumor cultured in vivo [3, 4], has been used for the maintenance of gastrointestinal organoid cultures [5, 6]. While highly effective for culture of organoids, Matrigel is xenobiotic and may contain excessive growth factors due to its tumor origin [7]. As the ECM microenvironment significantly affects cell and tissue development, the organoid field continues to explore ECM scaffolds which improve the physiological relevance of ECMs for organoid culture [8].

Synthetic ECMs are often utilized in favor of Matrigel, with polyethylene glycol (PEG)-based and polyacrylamide-based gels being the most common [8, 9]. Synthetic gels are attractive alternatives because their components are defined, and their physical and mechanical properties are tunable. However, they often require significant chemical functionalization for bioactivity and may not accurately recapitulate the tissue microenvironment. The use of tissue-derived ECMs can mimic the tissue-specific microenvironment, and the use of species-matched ECMs may alleviate the potential xenobiotic effects of Matrigel. ECM-derived products and hydrogels are typically isolated by decellularization of the tissue of interest. Decellularization refers to the removal of cellular components through chemical, enzymatic or mechanical means, leaving only the intact extracellular structure [10–12]. These techniques have been thoroughly described, as decellularized tissue is used for regenerative medicine applications such as grafting of the skin or heart [13, 14]. While decellularized tissue hydrogels reproduce the ECM microenvironment, they often fail to match the mechanical properties of the native tissue [15, 16]. Tissue-specific effects of decellularized tissue on cell development have been reported. Notably, ECM materials from decellularized tissues of different porcine organs were found to induce tissue specific gene expression of mesenchymal stem cells [17].

More recently, decellularization techniques have been applied for the development of tissue-specific ECMs for organoid culture [18, 19]. In 2019, Giobbe and colleagues produced a hydrogel from decellularized porcine intestinal mucosa and submucosa, which was successfully used for culture of endoderm-derived human organoids including intestinal, gastric, hepatic, and pancreatic organoids [20]. Since then, other ECM hydrogels have been reported as compatible for organoid growth. Kim and colleagues also developed ECM hydrogels from porcine intestine and stomach that were compatible with culture of human gastrointestinal tissue-derived organoids [21]. Decellularized bovine lung tissue was suitable for culture of human lung organoids and successfully retained patient specific morphology for personalized disease modeling [22]. ECM hydrogels from human tissue used for organoid culture have also been reported, though less commonly, due to limited access to tissue. Human mammary organoids were cultured in an ECM derived from human breast tissue, and cholangiocyte organoids were viable in liver-derived ECM hydrogels, though proliferation rates were reduced [23, 24]. Brain organoids were cultured in ECM derived from decellularized human brain, which improved neurogenesis compared to organoids in Matrigel [25]. Human endometrium-derived ECM was also used for culture of human endometrial organoids, and these organoids and were more like native endometrial tissue than those cultured in Matrigel when compared by proteomic analysis [26].

Here, we report the development and characterization of a tissue- and species-specific ECM for culture of tissue-derived human gastric organoids (HGOs). This process involves decellularization and digestion of human stomach tissue. The ECM mixture, hgECM, polymerized into a viscoelastic gel with a fibrous microarchitecture. HgECM could also be supplemented with Matrigel for more robust formation of HGOs. We performed single cell RNA sequencing (scRNAseq) analysis of HGOs cultured in Matrigel or hgECM supplemented with Matrigel and found that culture in hgECM enhanced gene expression associated with growth and metabolism, and decreased expression associated with stress, epithelial-mesenchymal transition, and inflammation. To our knowledge, this is the first report on the use of human decellularized tissue-based hydrogel for the culture of gastrointestinal organoids.

## 2. Methods

### 2.1. Human tissues and organoids

All investigations were conducted in accordance with the ethical principles described in the Declaration of Helsinki and the institutional guidelines of our organizations. Human gastric tissue for preparation of organoids and decellularized tissue was obtained from the National Disease Research Interchange under exempt tissue protocol DB062615-EX (Institutional Review Board of Montana State University). Tissue samples were surgical discard tissues from bariatric surgeries or whole stomachs from transplant donors. Tissues were shipped on ice overnight, and processing began less than 30 h after procurement. HGOs were generated from gastric glands isolated from these tissues as described previously by our group [27]. Tissues used for preparation of hgECM and HGOs were macroscopically normal or had only mild to moderate inactive gastritis with no intestinal metaplasia.

### 2.2. Decellularization

Decellularized tissue was prepared using 6–15 g of gastric mucosa and submucosa. The gastric mucus was first removed from the luminal surface by gentle scraping with a glass slide. Next, the serosa and muscle layer were removed with surgical scissors and discarded. The remaining gastric tissue was sectioned in 2–5 cm^2^ pieces by scalpel. The gastric tissue pieces were treated with sterile distilled water containing a penicillin and streptomycin mixture (Gibco 15140–122), and amphotericin B (Cytiva, J220014), for 24 h, shaking on ice in a 4 °C refrigerator. The water was changed out 3–5 times over 24 h, to remove remaining blood and mucus. Next, the tissues were incubated in 0.1% Triton-X 100 (Fischer Bioreagents EP151-100) for 4 h at 4 °C, followed by a washing with water containing antibiotics and fungicide for one hour. For decellularization, the tissue was then treated with 2% sodium deoxycholate (Sigma Aldrich D6750-100) for 24 h at 4 °C, shaking. At 24 h, the treatment was removed, and the tissue was washed with water containing antibiotics for 24 h, replaced 3–5 times throughout. Next, the tissue was then treated with 20 U ml^−1^ DNase (Sigma Aldrich, DN25-100 mg) in 1 M NaCl (Fischer Chemical, 5271–500) and incubated for 3 h with gentle shaking at room temperature, to remove released cellular DNA. The decellularized tissues were then washed with water and antibiotics and fungicide for 72 h, on ice with gentle shaking at 4 °C to remove any remaining sodium deoxycholate or DNA. The water was replaced 3–5 times per day. This step was essential to remove any remaining sodium deoxycholate and DNA, as both inhibit organoid growth. After completion, hematoxylin and eosin (H&E) staining was performed to verify that no nuclei remained. Decellularized tissue was then freeze dried using a lyophilizer, with the optimal protocol as follows: primary drying at 100 mTorr, beginning with a 120 min hold at −40 °C, followed by 120 min holds at −30, −20, −10, and 0 °C, then a secondary drying at 100 mTorr and 0 °C for 60 min. Lyophilized decellularized tissue could be stored at −80 °C for at least two years with no reduction in polymerization or biocompatibility.

### 2.3. Hydrogel generation

Freeze-dried tissue was homogenized in sterile, ice-cold distilled water with penicillin, streptomycin and amphotericin B using a rotating tissue homogenizer at 30 000 rpm. The optimal concentration of freeze-dried material in water for gel formation and organoid growth was 5–7 mg ml^−1^. The decellularized homogenate was cleared of any remaining large pieces by centrifugation at 100 g for five minutes, then incubated with 6 mg ml^−1^ of porcine pepsin (Thermo Scientific J61679.06) in 0.1 M HCl (Fischer Scientific A144S-212) at room temperature with gentle rocking for 72 h. After pepsin digestion, the tissue homogenate was neutralized using 5 N NaOH (Fischer Chemical SS256-500), on ice, and the protein concertation was determined by Pierce^TM^ BCA protein assay (Thermo Scientific, 23227). This material was considered the finished hgECM and was used in all experiments. The collagens of the neutralized homogenate repolymerize into a hydrogel when incubated at 37 °C for 35–45 min. The human gastric hydrogel was stable at 4 °C for 4–6 months, and like other collagen-based hydrogels, could not be frozen and thawed.

### 2.4. DNA extraction

To confirm complete decellularization, DNA extraction was performed on hgECM from three donors, phosphate buffered saline (PBS) alone, and 1 × 10^6^ l-cells as a positive control. DNA extraction was performed using a E.Z.N.A Tissue DNA Kit (Omega BIO-TEK, cat# D3396-02) with proteinase K digestion, as instructed. Isolated DNA was quantified using a DeNovix DS-11+ spectrophotometer (Denovix, Inc).

### 2.5. Proteomic analysis

Decellularized tissue and hgECM from three paired donors were analyzed using Mass spectrometry (MS). Protein samples were reduced, alkylated, and purified by chloroform/methanol extraction prior to digestion with sequencing grade modified porcine trypsin (Promega, PRV5117). Tryptic peptides were then separated by reverse phase XSelect CSH C18 2.5 µm resin (Waters) on an in-line 150 × 0.075 mm column using an UltiMate 3000 RSLCnano system (ThermoFisher). Peptides were eluted using a 60 min gradient from 98:2 to 65:35 buffer *A:B* ratio (Buffer *A* = 0.1% formic acid, 0.5% acetonitrile, Buffer *B* = 0.1% formic acid, 99.9% acetonitrile). Eluted peptides were ionized by electrospray (2.4 kV) followed by mass spectrometric analysis on an Orbitrap Eclipse Tribrid mass spectrometer (ThermoFisher). MS data were acquired using the Fourier Transform MS analyzer in profile mode at a resolution of 120 000 over a range of 375–1400 m z^−1^ with advanced peak determination. Following high-energy collisional disassociation activation, MS/MS data were acquired using the ion trap analyzer in centroid mode and normal mass range with a normalized collision energy of 30%. Proteins were identified by database search using MaxQuant (Max Planck Institute) with a parent ion tolerance of 2.5 ppm and a fragment ion tolerance of 0.5 Da. Scaffold DDA (Proteome Software) was used to verify MS/MS based peptide and protein identifications. Protein identifications were accepted if they could be established with less than 1.0% false discovery and contained at least 2 identified peptides. Protein probabilities were assigned by the Protein Prophet algorithm [28]. The gene ontology (GO) term analysis for determination molecular functions of proteomes were annotated by Scaffold DDA with GO terms from National Center for Biotechnology Information (downloaded 8 May 2025) [29]. A principal component analysis (PCA) was performed on two matched decellularized tissues and hgECM samples using Scaffold DDA.

### 2.6. Scanning electron microscopy (SEM)

To prepare hydrogels for SEM, 500 µl of each gel were dispensed with a wide bore tip into a 24-well plate and polymerized for 4–8 h at 37 °C, then fixed with 1 ml of formaldehyde overnight. Polymerized hydrogels were then subjected to graded ethanol (EtOH) dehydration. The hydrogels were treated with 5 min washes in the following sequence: two washes with deionized water, two washes with 30% EtOH, and three washes each in 50%, 70%, 85%, 95%, 100% EtOH. After washing, the sample was submerged in 100% EtOH until critical point drying. Dehydrated samples were dried using a Leica EM CPD300 critical point dryer (Leica Microsystems). Samples were mounted into the dryer according to manufacturer instructions. The chamber and sample holder were immersed in 100% EtOH, and carbon dioxide was used as the transitional fluid. Drying was performed using the following settings: Auto mode enabled, slow carbon dioxide in, 360 s delay, exchange speed 1, 12 exchange cycles, slow gas out heating, and 20% gas out speed. Upon completion of critical point drying, the sample was stored in a desiccator until SEM imaging. The dried samples were loaded onto an aluminum sample stub using double sided carbon tape. Dehydrated hydrogels were sputter-coated with platinum in an Auto Fine Coater JEC-3000FC with Rotating Specimen Stage (JEOL Ltd). The sample sputter-coated with a chamber pressure <20 Pa using a current of 30 mA for 120 s, creating a platinum coating thickness of <100 nm. Following platinum coating, the hydrogels were imaged using a JEOL JSM-IT710HR field emission scanning electron microscope (JEOL Ltd). Images of Matrigel and hgECM were obtained with a probe setting of 35, and accelerating volt of 5 kV or 10 kV, respectively. Sample images were taken at ×10 000 and ×16 000 to assess surface morphology and ECM architecture. For image analysis, SEM images were assessed with the Fiji plugin bundle for ImageJ (National Institutes of Health). Collagen fiber diameters and Matrigel pores were measured using calibrated figures. Forty collagen fiber diameters were measured, and fourteen Matrigel pores were assessed.

### 2.7. Rheometry

Rheological properties of hgECM from three donors (5–6 mg ml^−1^) and two batches of Matrigel (10–12 mg ml^−1^) were measured using an AR-G2 or Hybrid HR 30 rheometer (TA Instruments) with a 20 mm parallel plate geometry (TA Instruments). 500 µl of either Matrigel or hgECM were polymerized in a 24-well plate at 37 °C overnight, then a portion of the solidified gel was removed using a scooping spatula and transferred to the rheometer. A series of rheometric tests were performed at room temperature in the following order: 1) flow sweep with a shear range from 1 to 200 s^−1^, 2) an amplitude sweep was performed at a constant frequency of 10 rad s^−1^ and an oscillation strain from 0.01 to 20%, and 3) a frequency sweep from 0.1 to 200 rad s^−1^. After polymerization, the storage modulus was determined using a frequency sweep from 0.1 to 200 rad s^−1^ . Viscosity was determined using differential shear rates from 1 to 200 s^−1^. Samples were allowed to recover for 10–15 min in between tests.

### 2.8. HGO culture

HGOs were cultured in L-WRN media, which contained Advanced Dulbecco’s Modified Eagle Medium (DMEM) (Gibco) with 10% fetal bovine serum, 2 mmol l^−1^ L-glutamine (Gibco), Penicillin-streptomycin (Gibco), 50 mg ml^−1^ gentamycin (Gibco), 0.25 µg ml^−1^ Amphotericin B (Gibco), 10 µM ROCK-inhibitor Y-27632 (Tocris Bioscience), 10 µM TGF-*β*-inhibitor SB431542 (Tocris Bioscience), with 50% conditioned media from L-cells which constitutively expressed Wnt3a, R-spondin 3, and Noggin, as described previously [5]. Every 5–7 d, HGOs were passaged 1:4 as follows: HGO patties were collected into cold PBS with penicillin and streptomycin by pipette scraping, then spun at 200 g. Matrigel pellets were incubated at 37 °C with 200–300 µl 0.25% trypsin and EDTA, followed by vigorous pipetting 40 times, then washed with 5 ml organoid wash media (advanced DMEM with penicillin and streptomycin), pelleted at 200 g, and resuspended in Matrigel or hgECM for re-plating.

### 2.9. HGO image analysis

For image analysis, organoids were imaged at 4X magnification using a Keyence microscope (Keyence). Four wells were imaged per condition at days 1, 3, and 5 after plating. Images were obtained from the center of the gel patty and approximately 2/3 above the bottom of the plate, and the same area was imaged each day. For analysis of organoid formation, all HGOs in focus were counted at day 5. For analysis of HGO size, the diameter of all HGOs in focus were measured using the image analysis software ImageJ. If HGOs were oblong, the greater diameter was measured. Growth experiments were repeated three times, with three different organoid lines and two hgECM donors used.

### 2.10. Culture of HGOs in hgECM

To evaluate HGO growth in hgECM, HGOs were cultured in Matrigel for 5–7 d, then passaged using trypsin. After trypsinization, the organoid fragments were diluted with washing media, then divided into three equal fractions and pelleted at 200 *g*. The pellets were then resuspended into Matrigel, hgECM, or hgECM + 25% Matrigel. For re-passaging of HGOs cultured in hgECM + 25% Matrigel, 3–4 wells of HGOs were passaged with trypsin as normal, with additional pipetting after trypsinization to fully break apart the hgECM patty. HGO fragments were then resuspended in 20–40 µl, for a final 10% dilution of hgECM + 25% Matrigel.

### 2.11. HGO viability

HGOs from two donors were cultured in 24-well plates as normal, then passaged and divided evenly into 150 µl of Matrigel, hgECM + 25% Matrigel, or hgECM alone, and plated in duplicate in a 96-well plate. After 72 h, cell culture media was removed, and 100 µl of the luminescent cell reporter assay, CellTiterGlo (Promega, G7570) was added. Luminescence was read after 15 min with an integration time of 1 s using a GloMax® Discover Microplate reader (Promega).

### 2.12. Preparation of HGOs for scRNAseq

Four lines of HGOs were passaged into six to eight wells of Matrigel and then cultured for 4–6 d until confluent. HGOs were then fragmented following typical organoid culture protocols described above. HGO fragments were separated equally into two, then each half was resuspended in five wells of Matrigel or hgECM with 25% Matrigel. HGOs were then cultured for four days, then HGOs were disassociated into single cells using trypsinization (Library A), or by cold protease digestion (Library B–D) [30]. For trypsinization, HGO pellets were washed in 1 ml of ice-cold PBS per well, then centrifuged at 200 g for five minutes. HGO pellets were then treated with 350 µl of 0.25% trypsin EDTA for ten minutes at 37 °C. Next the trypsin was diluted with 600 µl of cold DMEM, then the pipetted 100 times, to further disassociate the HGOs. The organoid cells were then washed with 5 ml of ice cold DMEM, then centrifuged for 7 min at 300 g. The organoid cells were then washed two additional times with ice cold DMEM, to remove any remaining Matrigel or hgECM. For cold protease digestion, HGOs were washed with 1 ml of ice-cold PBS per well, then incubated for one hour on a shaker at 4 °C in 2 ml of ice-cold PBS with 4 mg ml^−1^ native *Bacillus licheniformis* protease (Creative Enzymes NATE0633), 10 µg ml^−1^ DNase, and 5 mM CaCl_2_. The cell mixture was vortexed for 30 s every 20 min. After digestion, the cell mixture was filtered through a 60 µm filter, then washed twice with 5 ml of ice-cold DMEM and centrifuged at 300 *g* for 10 min. After disassociation, the total cell concentration and viability were determined using a hemocytometer and trypan blue staining. All cell mixtures used for single cell sorting contained between 50 000 and 1 million total cells and were at least 95% viable. HGO single cells were sorted and barcoded using a 10X Genomics Chromium Controller with Next GEM 3’ Reagent Kits v3.1. or a Chromium X with GEM-X Single Cell 3’ Reagent kits v4. See table 3 for additional information.

### 2.13. Single cell sequencing

Libraries were prepared according to 10X Genomics instructions using 10 000 cells (A) and (B) or 20 000 cells (C) and (D) per preparation. All libraries were sequenced at the Nevada Genomics Center on an Illumina NextSeq 2000 instrument at 30 000 reads per cell using the NextSeq 2000 P4 XLEAP-SBS Reagent Kit. Sequenced libraries were aligned using against the human GRCh38 reference using 10X Genomics CellRanger software. CellRanger v7.1.0 was used to align libraries A and B, and CellRanger v9.0.1 was used to align libraries C and D.

### 2.14. scRNAseq preliminary analysis

All further analysis was performed in R following previously established scRNAseq analysis pipelines [31]. The aligned libraries were imported into R using the Seurat package [32]. To exclude empty droplets and low-quality cells, events for which mitochondrial reads contributed to more than 15% or less than 0.5% of total reads were removed. Genes expressed in less than 2 cells were excluded from the analysis. Multiplet events were removed using scDblFinder, and the transcriptional profiles of remaining events were normalized and scaled using SCTransform. Using Seurat functions, a PCA dimensionality reduction was performed, and an elbow plot was generated to decide how many dimensions to use in further analyses. Events were clustered using 20 dimensions, and cluster markers were calculated. In each library, low-quality events clustered together. Low-quality clusters were defined by low total RNA counts, low total detected genes, and mitochondrial gene markers without corresponding marker gene evidence of high energy requirements. Low quality clusters were removed, and individual libraries were integrated, normalized, and scaled. PCA and Uniform Manifold Approximation and Projection (UMAP) dimensionality reduction was performed on the integrated library using 35 dimensions. The integrated library was re-clustered, and marker genes were calculated for each cluster. Clusters were annotated using the marker genes and expression plots of known gastric cell type markers (supplemental figure 3). Differential analysis between HGOs cultured with Matrigel and hgECM were performed using EdgeR [33]. Briefly, annotated groups for each organoid and ECM combination were pseudobulked and log scaled according to default EdgeR functions, and library sizes were normalized. Differentially expressed genes between Matrigel and hgECM cells were calculated after accounting for variation attributed to organoid line and cell annotation type using the quasi-likelihood method. Results were filtered to retain genes with an adjusted *p*-value of less than 0.05. Pathway analysis was performed by using the EnrichR package [33] to predict Hallmark pathway enrichment using statistically significant genes differentially expressed in hgECM or Matrigel HGOs.

## 3. Results

### 3.1. Generation of HgECM hydrogel

**Figure 1.**
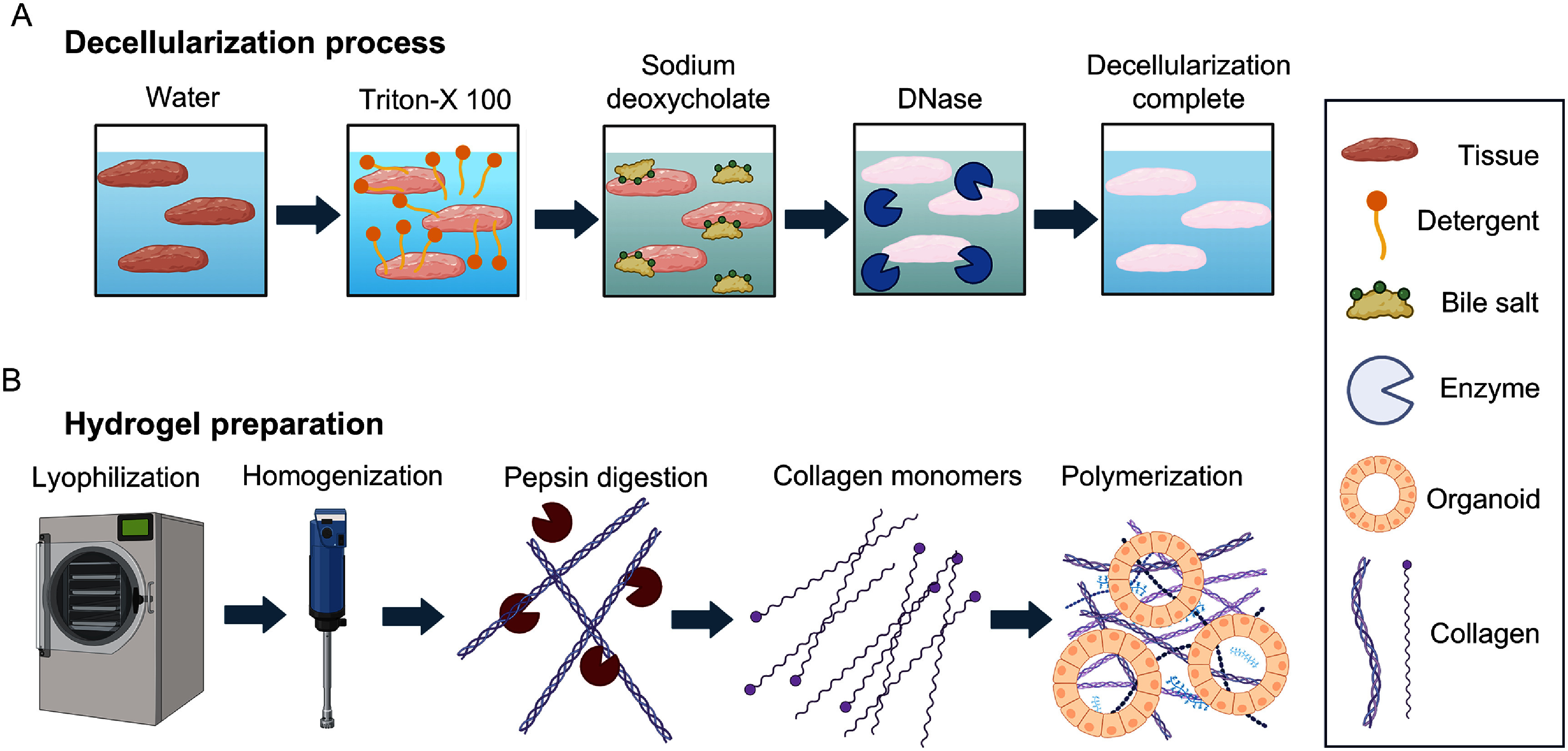
Graphical overview of human gastric extracellular matrix (hgECM) preparation for culture of human gastric organoids (HGOs). (A) To decellularize the human gastric epithelium and lamina propria, tissue pieces were incubated in sterile distilled water for 24 h, followed by permeabilization with triton X-100 for 4 h. Next, decellularization was performed with 2% sodium deoxycholate at 4 °C for 24 h, then the tissue was washed with sterile distilled water, and released DNA was removed by treatment with DNase I. Decellularized tissue was then washed in water at 4 °C for 72 h to remove remaining DNA and cellular contaminants. (B) For hydrogel preparation, the decellularized tissue was lyophilized, then homogenized into distilled water with antibiotics and fungicide at a concentration of 5–6 mg tissue ml^−1^ . Collagens in the ECM mixture were digested into monomers with 6 mg ml^−1^ porcine pepsin and 0.1 M HCl for 72 h. The digested ECM mixture was then neutralized and repolymerized with HGO fragments at 37 °C. Created in BioRender. Cherne 2026 https://BioRender.com/ekaikx9, https://BioRender.com/qjg3mtu.

**Figure 2.**
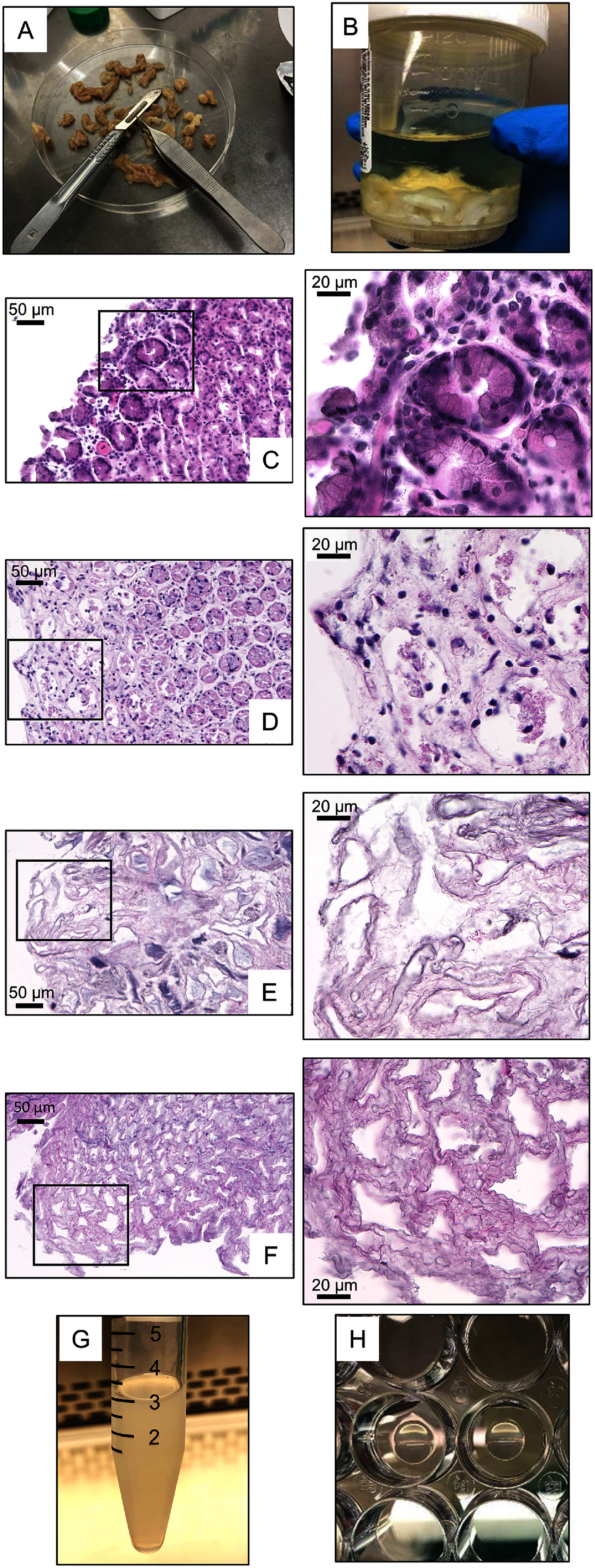
Decellularization of human gastric tissue and preparation of hgECM. (A) Human gastric epithelium and lamina propria sectioned into pieces for decellularization. (B) Gastric epithelium and lamina propria were colorless after completion of the decellularization process (C)–(F) Hematoxylin and eosin (H), (E) differential staining of gastric tissue demonstrating the removal of nuclei after the decellularization process. (C), (H), (E)staining of gastric tissue before decellularization (D) after treatment with water, (E) detergents and (F) after decellularization was complete. (G) Unpolymerized hgECM after digestion with pepsin and neutralization. (H) Polymerized patties of hgECM. Data are representative of three independent experiments.

Human gastric tissue was procured from resected surgical tissue from bariatric procedures, or by organ donation. The decellularization protocol, depicted in figure 1, was modified from a previously published protocol for decellularization of porcine intestine [34]. To ensure that the generated hydrogel would match the extracellular environment of the gastric epithelium, the serosa and muscle layer was removed from the tissue section, leaving only the tunica mucosa and tunica submucosa. This tissue was then cut into 2–5 cm^2^ pieces, so that the decellularization treatments could effectively diffuse through entire tissue (figure 2(A)). After decellularization, the pinkish to brown tissue (figure 2(A)) became white and transparent (figure 2(B)). To assess the quality of the decellularization protocol, we performed a differential stain, H&E, after each step in the decellularization process, to identify remaining nuclei (figure 2(C)–(F)). To begin decellularization, tissue pieces were treated with deionized water supplemented with antibiotics, to remove blood, mucus, and other impurities. Treatment with water initiated cell lysis in the tissues, but some nuclei remained (figure 2(D)). Next, the tissue was treated with 0.1% triton-X 100, to permeabilize the cells, then with 2% sodium deoxycholate for 24 h, to complete the decellularization. After treatment with detergents, no intact nuclei remained, but some pockets of DNA were still observed, visualized by purple hematoxylin staining (figure 2(E)). To remove the DNA released by cell lysis, tissues were then treated with DNase I. Removal of this extracellular DNA was essential to prevent cell stress during organoid culture, mediated by damage associated molecular pattern (DAMP) receptors [35, 36]. Finally, detergents and remaining cellular contaminates were removed by washing in deionized water with antibiotics for 72 h. H&E staining showed the dark purple areas of DNA visible in figure 2(E) were mostly removed after this final wash (figure 2(F)). Notably, the glandular architecture of the tissue was preserved after decellularization (figure 2(F)). Preservation of the ECM architecture suggests many of the native biological and mechanical properties of the tissue are maintained [12].

After decellularization, the tissue was flash frozen in liquid nitrogen and then lyophilized. Freeze-dried decellularized tissue could be used immediately to prepare hydrogels or could be stored at −80 °C. Hydrogels were successfully generated from this lyophilized tissue as long as two years after storage. For hydrogel preparation, the decellularized tissue was homogenized into water with antibiotics and fungicide. The optimal protein concentration for full polymerization was 5–6 mg ml^−1^. Due to the toughness of the decellularized tissue, some protein components remained unhomogenized, therefore, the final concentration was confirmed biochemically, rather than by dry tissue weight. After homogenization, debris was removed by centrifugation, leaving a white, opaque mixture of ECM components (figure 2(G)). Collagens within the homogenate were then digested for 72 h with porcine pepsin. After digestion, the mixture was cooled to 4 °C, then the pH was adjusted to neutral. To confirm the decellularization protocol depleted hgECM of DNA, we performed DNA extraction on hgECM from three donors in parallel with a PBS control, and 1 × 10^6^ l-cells, as a positive control. DNA was undetectable in two of the three samples and was present at 0.64 µg µl^−1^ in the third sample (table 1). The unpolymerized hydrogel could then be polymerized into a gel by incubation at 37° C for 35–45 min or stored at 4 °C for 2–4 months. After four months, hgECM did not fully polymerize. HgECM could be polymerized into 30–40 µl domes bound to tissue culture-treated polystyrene plates, but hgECM patties were prone to detachment if agitated (figure 2(H)).

**Table 1.** DNA concentration of hgECM.

Sample	DNA concentration (µg µl^−1^)
PBS (−)	0
HgECM 1	0
HgECM 2	0.647
HgECM 3	0
L-cell (+)	19.26

### 3.2. Proteomic analysis of hgECM reveals a sparse mixture dominated by collagens

**Figure 3.**
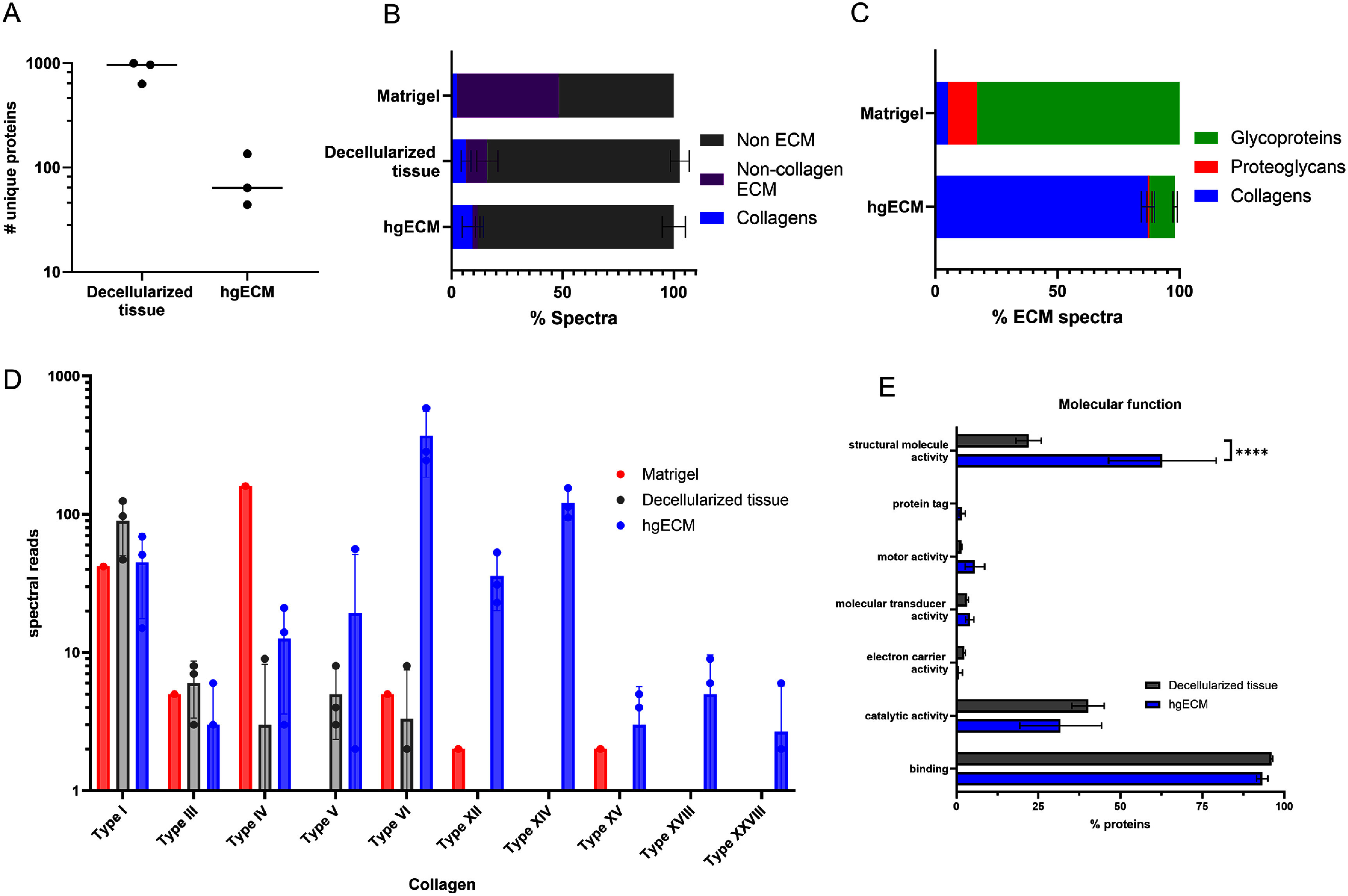
Proteomic analysis of decellularized tissue, human gastric extracellular matrix, and Matrigel. Proteomic composition of decellularized tissue and matched hydrogels from three donors were determined using tandem mass spectrometry. Proteins were identified using at least 2 peptides and 1% FDR. (A) Unique proteins identified in decellularized tissue and hgECM. (B) Comparison of ECM and non-ECM components of Matrigel, decellularized tissue, and hgECM, displayed as percentage of total spectral reads [37]. (C) ECM composition of the Matrigel and hgECM proteome, displayed as percentage of total spectral reads. (D) Collagen types detected in Matrigel, decellularized tissue, and hgECM, displayed as total spectral reads. (E) Gene ontology (GO) term enrichment analysis of molecular function of decellularized tissue and hgECM proteomes. **** = *P* <0.0001, determined by Šídák’s multiple comparisons test.

We next determined the protein composition of decellularized tissue and hgECM by performing tandem mass spectroscopy analysis of decellularized tissue and hgECM from three matched donors. The hgECM proteome was lower in complexity by tenfold compared to whole decellularized tissue, with 81 ± 47 unique proteins identified, compared to 865 ± 203 unique identified in the decellularized tissue (figure 3(A)). We also compared the protein composition of decellularized tissue and hgECM with a publicly available proteomic dataset of Matrigel [37]. When percentage of spectral reads for collagens, other ECM components such as proteoglycans and glycoproteins, and non-ECM components were compared, Matrigel was 51.7% non-ECM proteins, while decellularized tissue and hgECM were predominantly non-ECM proteins, at 86.7 ± 4.2% and 88.3 ± 5.1%, respectively (figure 3(B)). Non-ECM proteins identified in hgECM and decellularized tissues were mostly contaminants from cell lysis (keratins, myosin, and metabolic enzymes) and secreted components of the stomach (digestive enzymes and mucins). No growth factors were detected in hgECM. Compared to decellularized tissue, hgECM was enriched for collagens and reduced non-collagen ECM components (figure 3(B)). The most common non-collagen ECM components identified in decellularized tissue were the basement membrane proteoglycan, perlecan, and the glycoprotein, tenascin-X (table 2). The most common non-collagen ECM proteins in hgECM were fibronectin and fibrillin-1 (table 2). Matrigel, which is predominantly laminin, was predictably 45.8% non-collagen ECM (figure 3(B)).

**Table 2.** Top ten most detected ECM proteins of human gastric decellularized tissue, human gastric ECM, and Matrigel.

#	Decellularized tissue	hgECM	Matrigel
1	Collagen alpha-3(VI) chain	Collagen alpha-1(I) chain	Laminin subunit alpha-1
2	Collagen alpha-1(XIV) chain	Collagen alpha-2(I) chain	Laminin subunit gamma-1
3	Collagen alpha-1(VI) chain	Fibronectin	Laminin subunit beta-1
4	Collagen alpha-2(VI) chain	Collagen alpha-3(VI) chain	Perlecan
5	Perlecan	Collagen alpha-1(III) chain	Collagen alpha-2(IV) chain
6	Tenascin-X	Fibrillin-1	Fibronectin
7	Fibronectin	Collagen alpha-2(IV) chain	Collagen alpha-1(IV) chain
8	EMILIN-1	Collagen alpha-2(V) chain	Laminin subunit alpha-5
9	Collagen alpha-5(VI) chain	Collagen alpha-1(V) chain	Collagen alpha-1(I) chain
10	Collagen alpha-1(XII) chain	Bone marrow proteoglycan	Laminin subunit beta-2

Basement membrane proteins, such as laminin and collagen type IV, are essential for organoid culture anchoring epithelial cells to the matrix and instructing cell polarity [38]. We therefore compared basement membrane composition between hgECM and Matrigel. Basement membrane components were less common in decellularized tissue and hgECM, compared to the laminin-rich Matrigel. No laminin was detected above the limit of detection in hgECM, though we identified nine unique laminin monomers in decellularized tissue, representing the alpha, beta, and gamma subunits of the laminin trimer (table 2) [39]. Though laminin was undetectable in hgECM, other basement membrane components were identified at significant quantities, particularly collagen IV, and collagen VI [40, 41]. This matched the predicted composition of each hydrogel, as whole tissue tends to be collagen rich, while the EHS tumor, from which Matrigel is derived, secretes excessive amounts of basement membrane components [4]. The top ten most identified ECM proteins in decellularized tissue, hgECM, and Matrigel, calculated by average spectral read, are reported in table 2. The complete proteomics data set is available via the PRIDE database under the identifier PXD081816.

The ECM composition of hgECM was predominantly collagen (figure 3(C)). As collagen composition is tissue specific, we investigated the unique collagen composition of hgECM [40]. Eighteen unique collagen monomers from ten collagen types were identified in decellularized tissue and hgECM (figure 3(D)). We identified type I collagen as the predominant fibril forming collagen detected in all three samples [40]. This was expected, as collagen type I is the most abundant collagen type in mammals and within connective tissues [42]. Collagen type IV, a basement membrane component, was the most abundant in Matrigel, but could also be found in hgECM and decellularized tissues (figure 3(D)) [40]. The most common collagen detected in hgECM was type VI (figure 3(D)), a network-forming collagen which supports attachment of the ECM to the basement membrane [40, 41, 43]. The second most detected collagen type in hgECM was type XIV, classified as a fibril associated collagen with interrupted triple helices (FACIT) [40]. HgECM was also enriched for other non-classical collagen types such as type XII, another FACIT, and the basement membrane component, collagen type XVIII (figure 3(D)) [40, 44]. Interestingly, the hgECM proteome had only mild donor-to-donor variability. ECM composition was consistent across hgECM samples, as was composition (figure 3). A PCA analysis of matched decellularized tissue and hgECM from two donors revealed minimal variability between hgECM, compared to the variability of the decellularized tissue (supplementary figure 1).

Last, we performed a gene ontology (GO) analysis of matched decellularized tissue and hgECM, to determine if hgECM was enriched for ECM components after processing. (figure 3(E)). When molecular functions were compared, hgECM had a significantly higher percentage of proteins predicted to have structural molecule activity compared to decellularized tissue (figure 3(E)). All other molecular functions were detected were not significantly different between decellularized tissue and hgECM. This analysis also revealed evidence of cellular contamination, with 45% of identified proteins in decellularized tissue and 30% of identified proteins in hgECM were related to catalytic activity. In summary, hgECM maintained tissue-specific features of decellularized human gastric tissue and possessed a unique collagen-dominant composition distinct from Matrigel. Despite minimal laminin detection, hgECM maintained a subset of basement membrane components suitable for organoid applications.

### 3.3. Microarchitecture of polymerized hgECM and Matrigel

**Figure 4.**
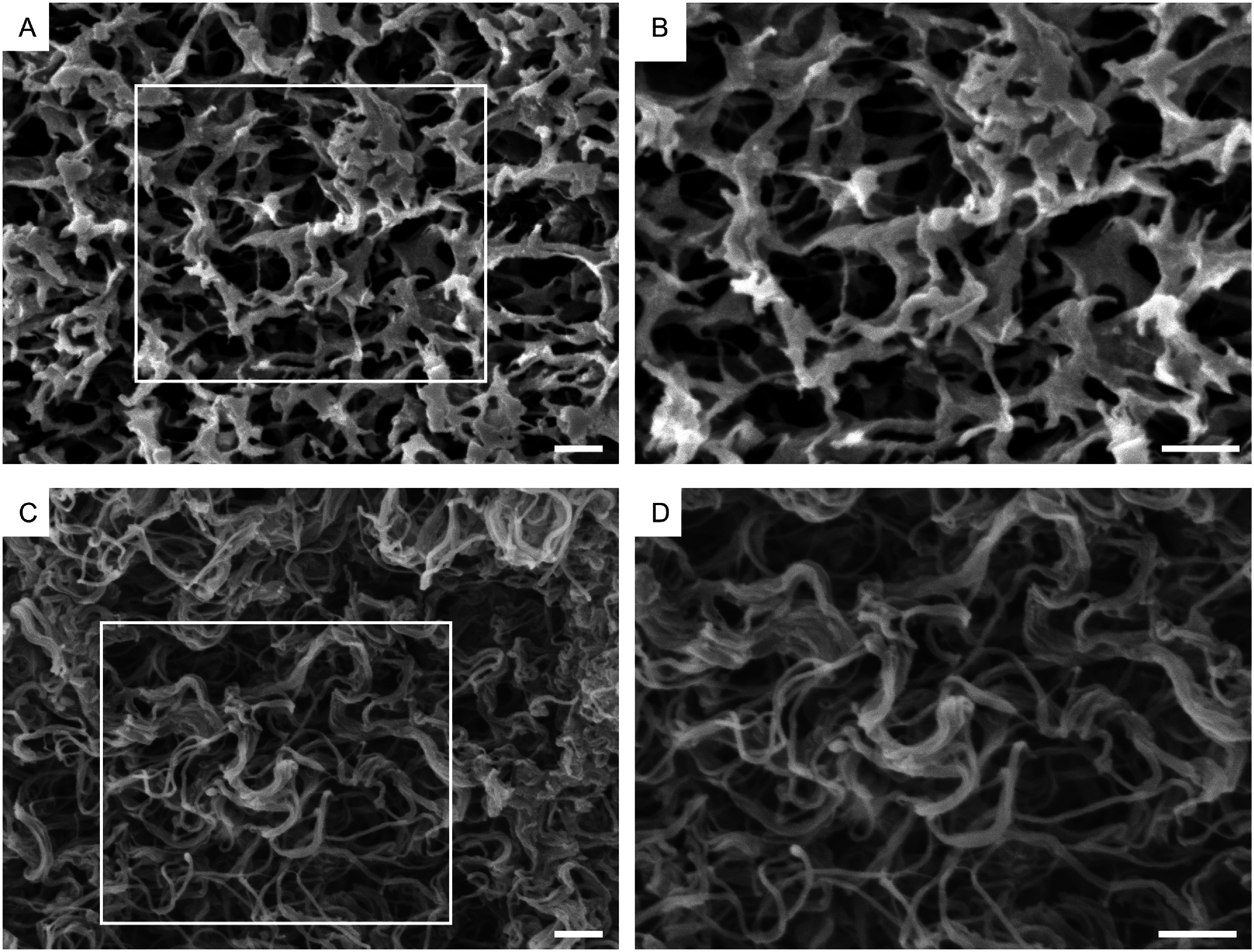
Scanning electron micrographs of polymerized Matrigel (A-B) and hgECM (C-D). Matrigel and hgECM were polymerized, dried then sputter-coated with platinum, then imaged at 10 000X and 16 000X. scale bar = 1 µm. One representative sample of Matrigel and hgECM.

The microarchitecture of ECM scaffolds can have broad cellular effects, so we also investigated the structural features of polymerized hgECM and Matrigel using SEM [45]. One representative sample was used for both hgECM and Matrigel. Polymerized HgECM was fibrous, typical of hydrogels rich in structural collagens, and did not have obvious pores (figures 4(A) and (B)) [46–48]. Interestingly, the hgECM fibers had a disorganized, wavy appearance (figures 4(A) and (B)). Fibers were also intermittently observed in curved bundles, ranging from 100 to 400 nm in width. The average fiber diameter of hgECM was approximately 83.2 nm ± 22.5 nm. The structure of Matrigel was distinct from hgECM, befitting its dissimilar proteome. Contrary to hgECM, Matrigel was not fibrous, but had a mesh-like, porous appearance (figures 4(C) and (D)), similar to other tissue-derived basement membrane scaffolds [49]. This was also expected, as Matrigel is a basement membrane mixture of laminin with minimal structural collagens [4]. The average pore size of Matrigel was 193 nm^2^ ± 94.8 nm^2^. Overall, hgECM and Matrigel had marked structural differences which reflect their distinct biochemical compositions.

### 3.4. Rheological characteristics of hgECM

**Figure 5.**
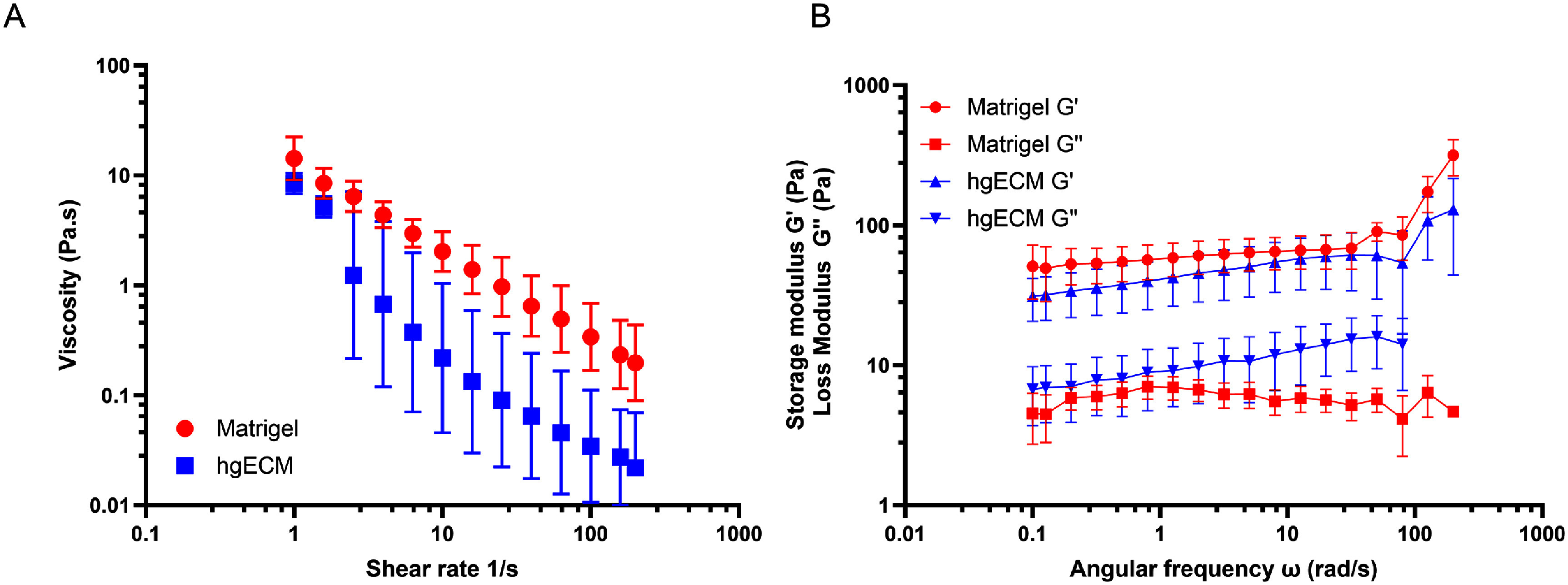
Rheological characteristics of hgECM and Matrigel. HgECM and Matrigel were polymerized at 37 °C overnight, then the material properties were analyzed at ambient temperature using a parallel plate rheometer. All frequency-dependent measurements were performed within the linear viscoelastic region, which was determined by amplitude sweep. (A) Viscosity was determined as a function of shear rate using a flow sweep test from 2 to 150 s^−1^. (Matrigel *n* = 5, hgECM *n* = 4), (B) Frequency-dependent storage (*G*′) and loss (*G*″) moduli of Matrigel and hgECM, determined by a frequency sweep from of 0.1 to 200 rad s^−1^ (Matrigel *n* = 4, hgECM *n* = 3).

We next compared the viscoelastic properties of hgECM and Matrigel, using hgECM prepared from three tissue donors and two batches of Matrigel. For each hydrogel, the protein concentrations used for organoid culture were tested (∼5 mg ml^−1^ hgECM and ∼12 mg ml^−1^ Matrigel). We assessed the material properties of hgECM and Matrigel after polymerization using a rotational rheometer with parallel plate geometry. First, viscosity was measured as a function of shear rate using a flow sweep test ranging between 1 and 200 s^−1^. Both materials were more viscous than water (viscosity of water = 10^−3^ Pa·s) [50]. The viscosity of both Matrigel and hgECM exhibited a negative slope, indicating shear-thinning behavior (figure 5(A)). The viscosity of hgECM was significantly lower than Matrigel, with hgECM viscosity typically 2–3 times lower than Matrigel over a shear range of 0.1–200 s^−1^.

The storage (*G*′) and loss (*G*″) moduli of hgECM and Matrigel were determined using a frequency sweep of 0.1–200 rad s^−1^ within the linear viscoelastic region (figure 5(B)). Storage modulus (*G*′) and loss modulus (*G*″) of each hydrogel remained constant at low frequencies, and *G*′ remained above *G*″, indicating elastic gel behavior. The storage modulus of both hydrogels increased at higher frequency (>50 rad s^−1^), a stiffening behavior common of fibrous biopolymers [51, 52]. To compare viscoelasticity, tan *δ* of Matrigel and hgECM were calculated using *G*′ and *G*″ (*G*″/*G*′) at *ω* = 1 rad s^−1^. Tan *δ* of both hydrogels were below one, indicating solid gel behavior (supplementary figure 2). Tan *δ* of Matrigel was greater, at 8.67 × 10^−2^, compared to the tan *δ* of hgECM was determined to be 2.01 × 10^−1^ (supplementary figure 2). We assumed both materials to be incompressible soft hydrogels, so Young’s modulus was calculated at 1 rad s^−1^ by *E* = 3*G*′. Young’s modulus of Matrigel was greater, at 175.9 Pa, compared to hgECM, 126.9 Pa, indicating Matrigel is more resistant to elastic deformation [53]. Rheological properties were more variable between hgECM batches than Matrigel batches, but this was likely caused by protein concentration, not composition, as different hgECM batches prepared from the same donor tissue had comparable variation. Overall, these rheological characteristics demonstrated hgECM retains the viscoelastic characteristics of a stable hydrogel, but with lower viscosity and less solid-like behavior than Matrigel.

### 3.5. HgECM is compatible for HGO culture

**Figure 6.**
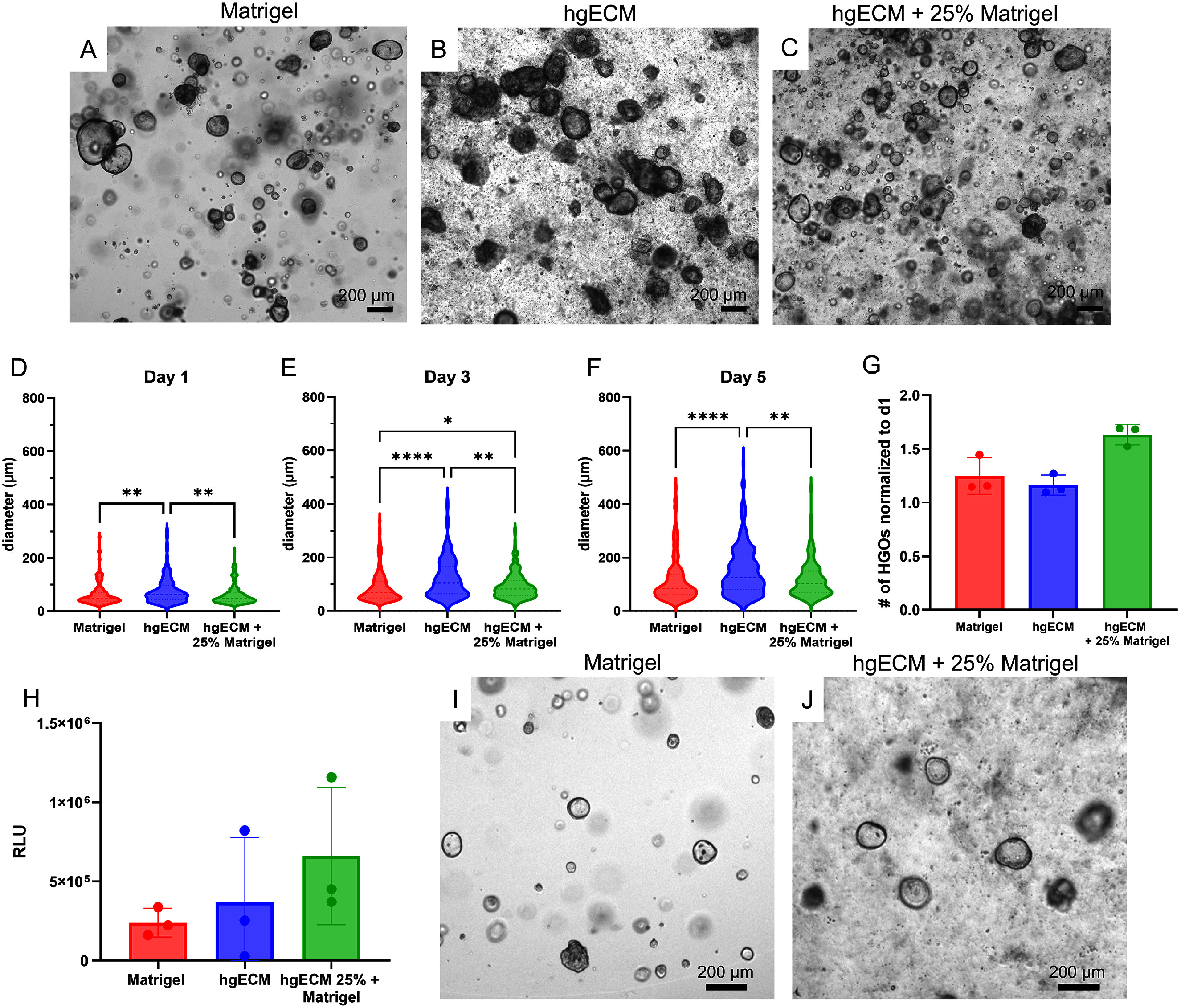
Human gastric extracellular matrix hydrogel supports growth of HGOs. HGOs were cultured in Matrigel for 4–7 d, then passaged into Matrigel, hgECM or hgECM supplemented with 25% Matrigel for 5 d. (A)–(C) Representative images of HGOs cultured in (A) Matrigel, (B) hgECM, or (C) hgECM + 25% Matrigel for 5 d. (D)–(F) HGOs were imaged 1, 3, and 5 d after plating, then the diameter of all HGOs in four images were measured using the image analysis software ImageJ. Representative data of one matched experiment. The diameter of all HGOs in focus were measured. Kruskal-Wallace test * = *P* < 0.05, ** = *P* < 0.01, *** = *P* < 0.001, **** = *P* < 0.0001. (G) The total number of HGOs identified at day five in each hydrogel normalized to the day one HGO count (*n* = 3). (H) HGOs were passaged into each hydrogel, then presence of metabolically active cells was compared using a luminescent cell reporter after three days of culture (*n* = 3). HGOs were cultured in hgECM with 25% Matrigel for one week, then HGO fragments were recovered from the gel by trypsinization. Fragments were re-embedded in either Matrigel (I) or hgECM + 25% Matrigel (J) for five days before imaging, a representative paired experiment is shown.

We next determined whether hgECM could be utilized for culture of HGOs. HGOs were passaged following our standard protocol [27, 54], and the cell pellet was resuspended into either Matrigel, hgECM, or hgECM supplemented with 25% Matrigel. After 45 min, patties of gastric ECM were gelled, L-WRN media was added, and gastric organoid formation and growth was compared by image analysis over five subsequent days (figures 6(A)–(C)). The HGOs in hgECM tended to grow to a larger size (figures 6(D)–(F)) compared to the other ECMs. However, fewer HGOs formed in hgECM after passaging compared to those in Matrigel, while the addition of 25% Matrigel to the hgECM increased the number of formed organoids (figure 6(G)). While image analysis was performed between 1 and 5 d, HGOs were cultured as long as 9 d in hgECM or hgECM + 25% Matrigel. However, as hgECM was a softer material, beyond five to seven days, larger HGOs near the bottom of the hgECM gel patty would occasionally collapse and form monolayers. A cytotoxicity assay which measures total cells, CelltiterGlo, revealed a similar trend. After HGOs were seeded into each hydrogel in parallel, the greatest number of organoid cells detected in hgECM + 25% Matrigel, though this was not statistically significant (figure 6(H)). HGOs cultured in hgECM with 25% Matrigel could also be passaged into fresh hgECM with 25% Matrigel, or Matrigel alone, with no observed difference in morphology or growth (figures 6(I)–(J)).

### 3.6. scRNAseq of HGOs cultured in hgECM reveals increased expression of gastric epithelial markers associated with differentiation

**Figure 7.**
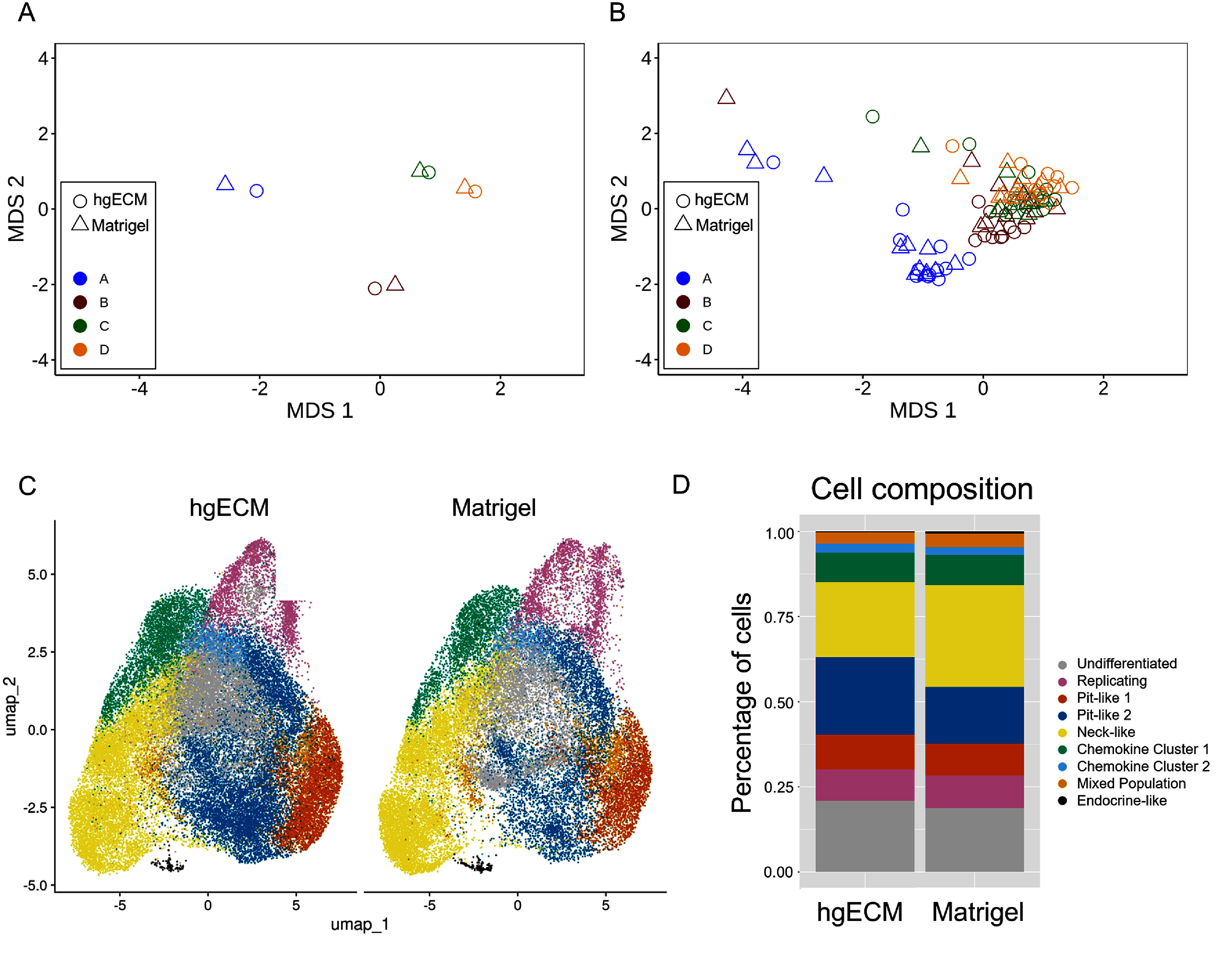
Single cell RNA sequencing of HGOs cultured in hgECM revealed no cell type differences, but a preference for mucus pit cells. HGOs were cultured in Matrigel for 5–7 d, then passaged into either Matrigel or hgECM supplemented with 25% Matrigel. After four days, HGOs were reduced to single cells enzymatically then barcoded and sequenced following 10X Genomics protocols. Eight libraries were prepared from four matched experiments, using four HGO lines and three hgECM donors. Each paired set of libraries are labeled A–D. (A) Multidimensional scaling (MDS) of each library. (B) Multidimensional scaling of each library and corresponding cell type. (C) Uniform Manifold Approximation and Projection (umap) of single cells recovered from hgECM and Matrigel. (D) Cell type composition of HGOs cultured in hgECM or Matrigel, depicted as percentage of total cells.

To determine the effect of culturing organoids in species- and tissue-specific hgECM, we performed scRNAseq of HGOs from four donors cultured in either Matrigel or hgECM supplemented with 25% Matrigel. Matrigel supplementation was chosen to enhance HGO formation, so that sufficient cell numbers could be recovered for sequencing. Paired experiments are referred to as libraries A–D. HgECM from three donors was used, with libraries B and D cultured with the same hgECM donor. Of note, the hgECM donor used for library C was *H. pylori* positive, and the HGOs in hgECM in library B were cultured in autologous hgECM (table 3). After culture for four days, HGOs were broken down into single cells using trypsin or by cold protease digestion, then barcoded using the standard 10X Genomics protocol. cDNA libraries were sequenced, and sequences were aligned to the human genome. Single cell profiles were analyzed using paired sample design, as each experiment used genetically identical samples exposed to two different conditions. After an initial exclusion of dead cells and duplicates, single gene expression analysis was performed using 70 220 cells total, with 40 998 recovered from culture in hgECM and 29 222 from Matrigel. Further details of each library are available in table 3. Multidimensional scaling (MDS) revealed that the greatest source of variation between libraries, with and without accounting for cell type differences, was the HGO donor (figures 7(A) and (B)). An initial clustering analysis revealed HGOs cultured in hgECM or Matrigel both predominantly contained cells with expression profiles comparable to mucus neck cells, mucus pit cells, and undifferentiated, replicating cells (figure 7(C)). Smaller cell populations included a cluster characterized by high expression of chemokines, and a cluster with a gene expression profile reminiscent of enteroendocrine cells due to high expression of somatostatin (*SST*) and ghrelin and obestatin prepropeptide (*GHRL*). The parameters used for cell annotations are presented as a dot plot featuring percent cell expression and average expression in supplementary figure 3. HgECM did not recover additional epithelial cell types, such as parietal cells. However, HGOs in hgECM had increased presence of mucus pit cells, while HGOs in Matrigel had greater preference for mucus neck cells (figure 7(D)). HGOs in Matrigel also had significantly higher expression of the chief cell marker *PGC*, however, no cell clusters were identified as chief cells. This is likely explained by the increase in mucus neck cells, which also express *PGC* [55].

**Table 3.** Donor information and experimental details of paired single cell RNA libraries.

	Library A		Library B		Library C		Library D	
ECM type	hgECM	Matrigel	hgECM	Matrigel	hgECM	Matrigel	hgECM	Matrigel
ECM donor	1	—	2	—	3	—	2	—
ECM sex	M	—	M	—	F	—	M	—
H. pylori status	Negative	—	Negative	—	Positive	—	Negative	—
Protein concentration (mg ml^−1^)	6.4	8–12	5.1	8–12	6.0	8–12	5.4	8–12
HGO line	Hu0043	Hu043	Hu044	Hu044	Hu010	Hu010	Hu007	Hu007
HGO sex	F	F	M	M	F	F	F	F
Passage	6	6	4	4	5	5	5	5
Autologous	No	—	Yes	—	No	—	No	—
Cells analyzed	2766	542	2629	2922	5926	8702	29 677	17 056
Library preparation	Next GEM 3′ Reagent Kit v3.1	Next GEM 3′ Reagent Kit v3.1	Next GEM 3′ Reagent Kit v3.1	Next GEM 3′ Reagent Kit v3.1	GEM-X 3′ Reagent kit v4	GEM-X 3′ Reagent kit v4	GEM-X 3′ Reagent kit v4	GEM-X 3′ Reagent kit v4

**Figure 8.**
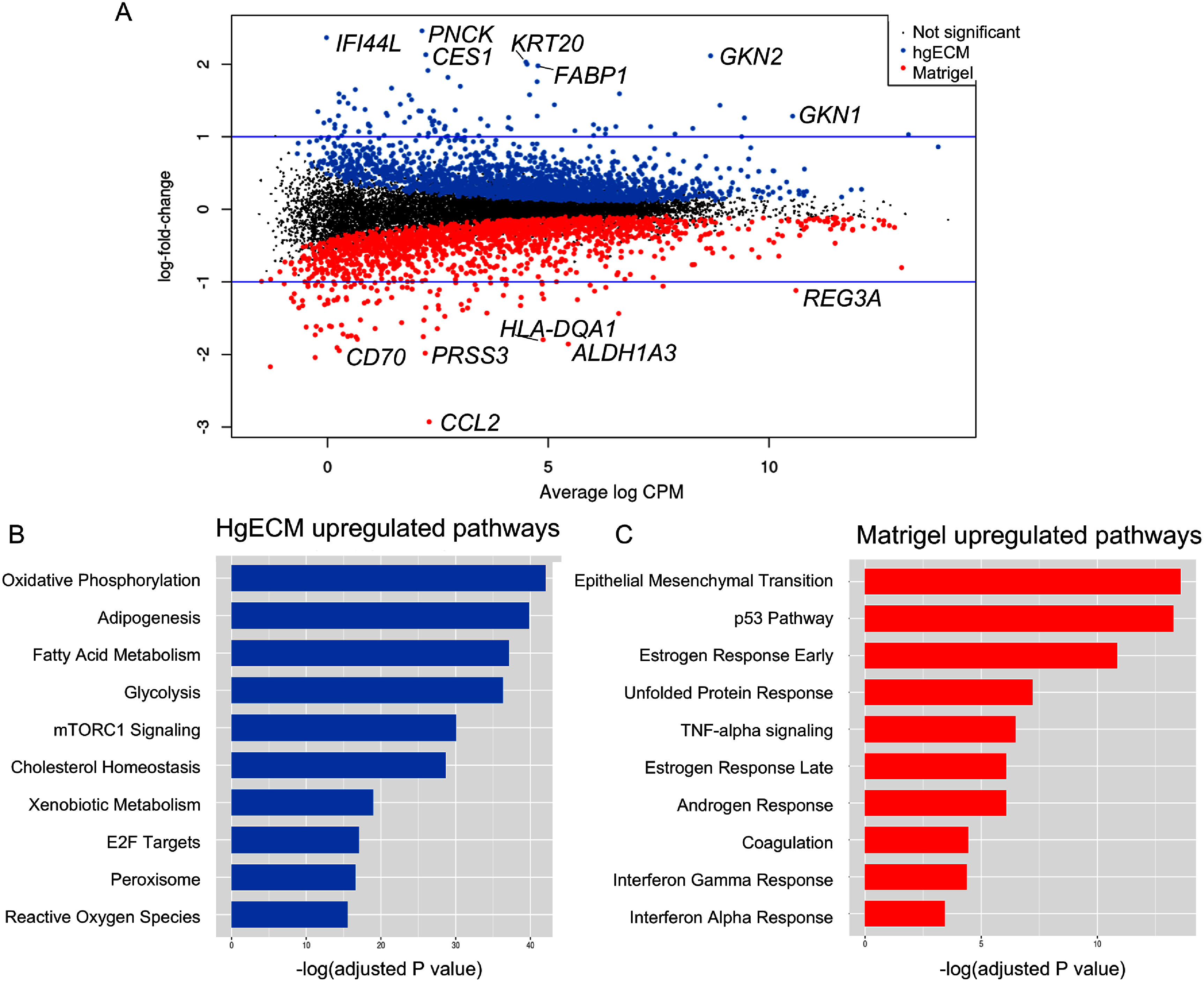
HGOs cultured in hgECM have reduced gene expression associated with stemness, stress, and inflammation compared to culture in Matrigel. Single cell libraries prepared from HGOs cultured in hgECM + 25% Matrigel or Matrigel for four days. Four paired experiments were pooled, and gene expression was compared. (A) Volcano plot of differentially expressed genes in HGOs cultured in hgECM + 25% Matrigel, or Matrigel, after accounting for variation within the ECM conditions, HGO donor variation, and cell types, with the *Y*-axis representing fold change of average gene expression, and the *X*-axis depicts counts per million (CPM). Each point represents one gene detected in the dataset. Blue points are expressed statistically higher in hgECM HGOs compared to Matrigel (*p* ⩽ 0.05), and red points are genes expressed statistically lower in hgECM HGOs compared to Matrigel (*p* ⩽ 0.05). Blue lines depict a 1 and −1 log-fold change. (B) Top 10 most significant Molecular Signatures Database Hallmark classifications of genes more highly expressed in (B) Matrigel and (C) hgECM.

We then investigated differential gene expression in response to hgECM, accounting for cell type and organoid donor-specific differences. In total, 2830 genes were significantly overexpressed in response to hgECM, and 2687 genes were significantly overexpressed in response to Matrigel. Results are visualized in figure 8(A) as a volcano plot. Of note, genes with the greatest fold increase in response to hgECM were pregnancy up-regulated nonubiquitous CaM kinase (*PNCK*), a stomach-specific cam-kinase, gastrokine 2 (*GKN2*), a stomach-specific factor with anti-inflammatory and anti-tumor activity [56], and *IFI44L* (Interferon-induced protein 44-like), an interferon-stimulated gene and a tumor suppressor [57]. Genes with the greatest fold change in the Matrigel group included *CCL2*, a proinflammatory chemokine that recruits monocytes and serves as a gastric cancer marker, *CD70*, an immune checkpoint molecule involved in epithelial-mesenchymal transition [58], and *HLA-DQA1,* a human class II major histocompatibility complex molecule involved in antigen presentation [59]. These identified shifts in cell composition and gene expression suggest HGOs cultured in hgECM had a more tissue-specific, differentiated state with reduced inflammatory signaling, compared to HGOs in Matrigel. The complete transcriptomics data set has been deposited to the Gene Expression Omnibus database, GSE303526.

### 3.7. HgECM reduces gene expression associated with stress, epithelial-mesenchymal transition, and inflammation

To identify differences in global gene signatures between organoids cultured in hgECM versus Matrigel, we performed a MSigDB Hallmark gene set expression analysis, accounting for specific cell types (figures 8(B) and (C)). HGOs cultured in Matrigel had significantly higher expression of gene sets associated with stress, epithelial-mesenchymal transition, allograft rejection, inflammation, and TNF-signaling via NF-*κ*B. In contrast, HGOs cultured in hgECM had significantly enhanced expression of cellular metabolism-associated gene signatures such as glycolysis, oxidative phosphorylation, mTORC1 signaling, and fatty acid metabolism.

**Figure 9.**
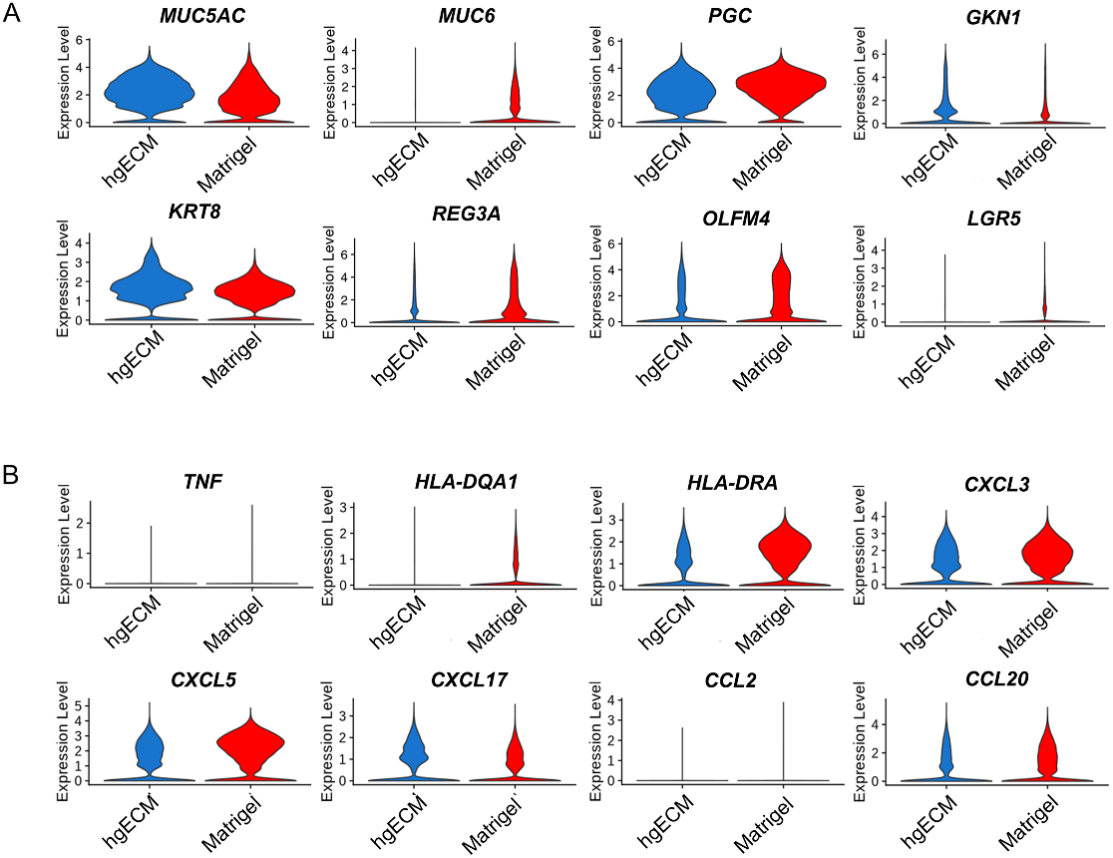
Differential expression of selected cell type, stem cell, and inflammatory genes induced by human gastric organoid culture in human gastric ECM or Matrigel. Four different HGOs lines were cultured in hgECM + 25% Matrigel or Matrigel for four days, then single cell libraries were prepared. Gene expression profiles were pooled from the four paired experiments and gene expression was compared. Pooled gene expression profiles of (A) selected genes for cell type, tissue specificity, and (B) inflammatory genes that were significantly differentially expressed between gel culture conditions (*p* ⩽ 0.05).

We then compared the expression of specific genes of interest, to better understand the effect of hgECM on HGO differentiation and homeostatic functions. We considered the effect of HGO culture in hgECM on cell differentiation. HGOs cultured in hgECM had increased expression levels of *MUC5AC*, while HGOs in Matrigel had significantly increased expression of the neck-cell marker *MUC6* (figure 9(A)), matching the increased presence of mucus pit cells in response to hgECM observed in the cell type analysis. *PGC* was also overexpressed in the Matrigel group, though no cell population was identified as chief cells (figure 9(A)). The expression of stem cell and proliferative cell markers *LGR5, OLFM4* (figure 9(A)) [60] was decreased in response to hgECM, suggesting a decrease in stemness towards differentiated cells. Towards this, cytokeratin 8 (*KRT8*), and gastrokin-1(GKN1), genes associated with mature gastric epithelium, were also upregulated in response to hgECM (figure 9(A)) [61–63]. Regenerating Islet-Derived Protein 3A (*REG3A*), was also overexpressed in response to Matrigel (figure 9(A)), *REG3A* is constitutively expressed in the gastrointestinal tract as a modulator of host-microbe interactions, and is overexpressed in gastrointestinal inflammatory diseases and cancer [64].

We further probed the observed upregulation of inflammatory pathways in response to Matrigel by investigating significantly overexpressed inflammatory genes. HGOs cultured in Matrigel increased expression of HLA genes *HLA-DQA1 and HLA-DRA* (figure 9(B)). Expression of HLA within the gut epithelium is highly suggestive of inflammation [59, 65]. The expression of inflammatory chemokines was also significantly upregulated in Matrigel cultured HGOs. The inflammation-associated C–X–C chemokines CXCL3 and CXCL5 were overexpressed in response to Matrigel, as well as the C–C group chemokines CCL2 and CCL20 (figure 9(B)). Only the chemokine CXCL17 was significantly overexpressed in response to growth in hgECM (figure 9(B)). Of note, CXCL17 is a chemoattractant for dendritic cells and macrophages and is specific to mucosal sites and is associated with anti-inflammatory effects and homeostasis [66, 67]. Overall, these findings indicate that hgECM may support a more homeostatic gastric epithelial phenotype, with improved differentiation and reduced stress and inflammatory responses.

## 4. Discussion

The ECM provides crucial biochemical and mechanical signals to the gastrointestinal epithelium which influence development and function [68–70]. Given the importance of these signals, the physiological relevance of gastrointestinal organoid models could be improved by integrating ECM scaffolds which recapitulate the species- and tissue-specific microenvironment. Towards this, we have developed and characterized an ECM hydrogel using decellularized human stomach which may improve HGO homeostasis and tissue-specific differentiation. To our knowledge, this is the first human-derived ECM scaffold specific for the culture of gastrointestinal organoids.

Hydrogels from decellularized tissues have been reported to induce tissue- and disease-specific cell and organoid differentiation and function [17, 71, 72]. The mechanisms underlying these effects are incompletely understood, but are likely driven by the unique combination of composition, structure, and material properties of each hydrogel. The ECM composition of hgECM was dominated by collagens. Collagen has a highly specific tissue distribution which may provide a physiologically relevant microenvironment for organoid growth [40, 73]. Towards this, in a study of decellularized pancreatic tissue hydrogels, collagen type V drove pancreatic islet organogenesis [71]. Collagen type I, common in loose connective tissue of the normal human gastric lamina propria and submucosa, was the most common structural collagen in hgECM [74]. Collagen type VI was the most abundant collagen in hgECM, matching the reported composition of decellularized porcine stomach [21, 75]. Type VI collagen is found throughout interstitial spaces and serves as part of the basement membrane, or as an anchor of the ECM to basement membrane [41, 76]. Collagen type VI and collagen type XII, both abundant in hgECM, contain non-collagen, globular domains which can direct tissue repair and homeostasis [77, 78]. The microarchitecture of ECM scaffolds, such as pore size and fiber size may also direct cell and organoid differentiation [75]. The effect of fiber characteristics on organoid differentiation and function is not well described, but shorter and stiffer collagen fibers can disturb cellular proliferation, migration, and differentiation [79, 80]. Polymerized HgECM had a fibrous structure in line with its collagen-rich composition, but had a loose, crimped microarchitecture. Notably, this structure was reminiscent of decellularized rat gastrointestinal tissue [81]. Overall, HgECM maintained a protein composition and microarchitecture similar to its tissue of origin, though further studies are needed to identify components of hgECM which impart differential HGO growth and gene expression.

The material properties of the ECM, including stiffness, viscoelasticity, and viscosity, are also tissue specific and can affect cell behavior at the single cell and tissue level [70, 82–84]. These properties can translate to disease pathologies, with stiffer ECM often associated with tumor microenvironments, malignancy, and chronic inflammatory disorders [85–87]. The mechanical properties of decellularized hydrogels also alter organoid differentiation and function [75]. Bejoy, *et al* reported the storage moduli of a hyaluronic acid-based hydrogel affected cell fate determination of brain organoids, with lower elastic moduli inducing forebrain development and a higher storage moduli favoring hindbrain development [88]. Notably, crypt formation of human intestinal organoids can be induced by matrix softening using photosensitive hydrogels [89]. Given HgECM was significantly less solid and viscous than Matrigel, the observed gene expression shift of HGOs in hgECM towards more mature gastric epithelium may be driven in part by these properties. Collectively, these findings suggest the conserved biochemical and material properties of hgECM may contribute to the improved HGO phenotype and gene expression profile.

HGOs cultured with hgECM had reduced gene expression associated with stress response, epithelial-mesenchymal transition, and inflammation markers, compared to HGOs in Matrigel. Considering the tumor origin of Matrigel, increased gene expression for epithelial-mesenchymal transition was not unexpected [4]. Of particular interest was the increased inflammatory profile. Matrigel is derived from whole, rather than decellularized tissue, so it is likely inflammatory factors would be present. Indeed, detection of inflammatory mediators in Matrigel including IL-23, C5a, endocan, and TIMP-1 by protein array has been previously reported [7]. The observation could also be explained by the greater material stiffness of Matrigel compared to hgECM, as stiffer ECM environments can induce inflammation [90]. This potential hyperinflammatory response to Matrigel should be considered when using HGOs for studies of homeostasis and inflammation. Our group has previously reported significant immune cell recruitment to HGOs in Matrigel, even at steady state [27]. Notably, HGO culture in Matrigel-supplemented hgECM enhanced gene expression of the recently described chemokine CXCL17 [67]. CXCL17 is exclusive to mucosal environments and is associated with anti-inflammatory effects and recruitment of immune cells at steady state [27]. Given these results, hgECM may serve as a more effective ECM scaffold for studies of HGO inflammation and immune recruitment.

Further optimization of hgECM would improve the utility of hgECM, as cellular contaminants remained within the hydrogel, and protein concentration was difficult to standardize. Additionally, HgECM prepared from individual donors may introduce variability. However, the proteome of hgECM was surprisingly similar donor-to-donor, and scRNAseq analysis identified greater variability between organoid donors than hgECM donors. In future studies, we will generate hydrogels from pooled donor tissues and process decellularized tissues as powders for improved standardization of concentration. Next steps will also explore the effect of long-term culture of HGOs in hgECM. There are also several limitations to our study. The availability of human tissue for material preparation is limited compared to non-human animals or synthetic materials. However, as the stomach is not used for organ transplantation, human gastric tissues are easier to obtain than other human tissues. Last, while our gene expression and growth analysis suggests HGOs in hgECM were better differentiated and under less stress, it is unclear if these results are caused by a specific effect of hgECM, or simply the reduction in adversarial effects of Matrigel. Further studies are needed to identify specific mechanisms responsible for hgECM-induced gene expression differences.

Our study introduces hgECM hydrogels from decellularized human gastric tissue, as highly compatible for culture of HGOs, addressing the need for physiological ECM scaffolds representative of the tissue from which they are derived. ScRNAseq analysis of HGOs cultured with hgECM demonstrated improved homeostatic functions and reduced inflammatory and stress responses, when compared to culture in Matrigel. Our method also has potential translational and personalized medicine implications, as HGOs can be cultured in autologous ECM. These results highlight the impact of the ECM microenvironment on HGO development and suggests hgECM may improve the physiological relevance of HGOs as models of physiology and disease.

## Data Availability

The data that support the findings of this study will be openly available following an embargo at the following URL/DOI: www.ncbi.nlm.nih.gov/geo/query/acc.cgi?acc=GSE303526 [91], www.ebi.ac.uk/pride/archive/projects/PXD081816 [92]. Supplementary Figures available at: https://doi.org/10.1088/1748-605X/ae943e/data1.

## Data transparency

Proteomics data is available via ProteomeXchange with identifier PXD066286; single cell RNA sequencing data has been uploaded to NCBI GEO, GSE303526, and is available using the following token: ubchumcmlxozvuh. Code for single cell RNA sequencing analysis will be deposited to GitHub.
